# Syndecan-1 Acts as an Important Regulator of CXCL1 Expression and Cellular Interaction of Human Endometrial Stromal and Trophoblast Cells

**DOI:** 10.1155/2017/8379256

**Published:** 2017-02-15

**Authors:** Dunja Maria Baston-Buest, Olga Altergot-Ahmad, Sarah Jean Pour, Jan-Steffen Krüssel, Udo Rudolf Markert, Tanja Natascha Fehm, Alexandra Petra Bielfeld

**Affiliations:** ^1^Department of Gynaecology, Center for Reproductive Medicine (UniKiD), Medical Faculty, University Duesseldorf, Moorenstr. 5, 40225 Duesseldorf, Germany; ^2^Department of Obstetrics, Research Lab/Placenta Lab, Research Center, Jena University, Building No. F2, Am Klinikum 1, 07747 Jena, Germany; ^3^Department of Gynaecology, Medical Faculty, University Duesseldorf, Moorenstr. 5, 40225 Duesseldorf, Germany

## Abstract

Successful implantation of the embryo into the human receptive endometrium is substantial for the establishment of a healthy pregnancy. This study focusses on the role of Syndecan-1 at the embryo-maternal interface, the multitasking coreceptor influencing ligand concentration, release and receptor presentation, and cellular morphology. CXC motif ligand 1, being involved in chemotaxis and angiogenesis during implantation, is of special interest as a ligand of Syndecan-1. Human endometrial stromal cells with and without Syndecan-1 knock-down were decidualized and treated with specific inhibitors to evaluate signaling pathways regulating CXC ligand 1 expression. Western blot analyses of MAPK and Wnt members were performed, followed by analysis of spheroid interactions between human endometrial cells and extravillous trophoblast cells. By mimicking embryo contact using IL-1*β*, we showed less ERK and c-Jun activation by depletion of Syndecan-1 and less Frizzled 4 production as part of the canonical Wnt pathway. Additionally, more beta-catenin was phosphorylated and therefore degraded after depletion of Syndecan-1. Secretion of CXC motif ligand 1 depends on MEK-1 with respect to Syndecan-1. Regarding the interaction of endometrial and trophoblast cells, the spheroid center-to-center distances were smaller after depletion of Syndecan-1. Therefore, Syndecan-1 seems to affect signaling processes relevant to signaling and intercellular interaction at the trophoblast-decidual interface.

## 1. Introduction

Successful implantation of a competent embryo into the human receptive maternal endometrium is a result of a complex network between hormones, growth factors, and chemokines [1]. All those factors are involved in the important early implantation process acting through their corresponding receptors and coreceptors by activating respective signaling pathways. Signaling pathways connect ligands with their receptors and their downstream targets inducing cellular functions, like gene transcription, cell proliferation, differentiation, or growth. Members of the mitogen-activated protein kinases (MAPKs) and wingless-Int1s (Wnt) signaling cascade are known to play a role at the human embryo-maternal interface regulating multiple steps in the embryo-maternal dialogue of implantation [2].

The MAPK pathway contains 3 key protein kinases and a phosphorylation cascade leading to signal transduction. Among them, the extracellular kinases 1/2 (ERK1/2) and the c-Jun N-terminal kinase (JNK) are the most prominent members of the MAPK pathway. MAPK signaling is involved in important cellular processes like proliferation, differentiation, cell fate, and apoptosis, which are all processes participating in human embryonic implantation [3, 4]. The huge Wnt signaling family plays a role in tissue differentiation, organogenesis, adhesion, tissue and cell polarity, migration, homeostasis, embryogenesis, implantation, and placentation [5–11]. Wnt ligands like Wnt 2, 3, 4, 5a, and 7a and frizzled (FZD) receptors, FZD1, FZD4, FZD6, and FZD10, are found to be expressed during the human and murine implantation and placentation process facilitating interactions like decidualization, endometrial gland formation, and embryo attachment [4, 10, 11]. Beta-catenin is the key mediator of the canonical Wnt pathway—mediating Wnt ligand to FZD receptor binding in its unphosphorylated form; upon phosphorylation it is degraded and in addition regulating cell adhesion by interaction with cadherins [12–14]. Bearing in mind that posttranscriptional and posttranslational regulation occur and phosphorylation status could not be observed on gene level, beta-catenin and FZD6 were found to be expressed uniformly in human proliferative and secretory endometrium [15]. Furthermore, nuclear factor kappa B (NF*κ*B) was found to be an important mediator of Syndecan-1 (Sdc-1) action during the tumour growth and invasiveness of endometrial cancer [16].

CXC motif ligand 1 (CXCL1) seems to be an important chemokine during embryonic implantation, since its expression increases significantly after treating decidualized (d) endometrial stromal (EnS) cells with trophoblast conditioned media or coculture with first-trimester trophoblast explants [17, 18]. We have previously shown the induction of CXCL1 expression by the embryo secreted factor IL-1*β* and its mediation by MAPK signaling and activation of NF-*κ*B in dEnS confirming the data of Issa et al. regarding regulation of CXCL1 via NF-*κ*B [19, 20]. CXCL1 acts through its receptor CXC motif receptor 2 (CXCR2) and the coreceptor Sdc-1 [21, 22]. Sdc-1 is one of 4 members of the human Sdc family and consists of one proteoglycan and several heparan sulfate chains. Sdcs are expressed on the surface of adherent and nonadherent cells and serve as coreceptors for various ligands like growth factors, proteases, protease inhibitors, chemokines, and cytokines mediating multiple physiological and pathological processes including regulation of cytoskeleton proteins, cell-cell and cell-matrix adhesion, and cell proliferation and inflammation. Besides their function via ectodomains, Sdcs might mediate cytoplasmic effects via intracellular domains [22]. Sdc-1 is upregulated during the secretory phase in the human menstrual cycle underlining an involvement in the preparation for embryo implantation [23]. Binding of CXCL1 on the cell surface enhances the molecules accessibility for its classical receptor CXCR2 in general and enables formation of a chemokine gradient involved in attraction of immune cells like monocytes [21, 24]. As published before, Sdc-1 seems to have a pivotal role for dEnS in regulating chemokine expression and apoptosis [25, 26].

Therefore, the aim of this study was to investigate the influence of Sdc-1 as a regulator of CXCL1 expression by mimicking embryo implantation via IL-1*β* with focus on the important signaling pathways MAPK and Wnt at the human trophoblast-decidual interface. As components of the Wnt pathway are known to be involved in adhesion, decidualization, and embryonic implantation we generated a new in vitro model based on two spheroids—EnS and trophoblast cells—to examine the interaction at the trophoblast-decidual interface.

The results provide a common link of Sdc-1's extra- and intracellular activities regarding the regulation of the CXCL1 secretion, the intracellular signaling via MAPK and Wnt pathway, and entry of human trophoblast into the endometrium, elucidating further the molecular events of the human peri-implantation period.

## 2. Materials and Methods

### 2.1. Cell Lines

In this study, two endometrial stroma cell lines were used: the human EnS cell line St-T1 known to be a reliable model for human endometrium and early decidua [27, 28] (a generous gift from Professor Brosens, University of Warwick, Coventry, UK) [29]. This cell line was further used in our laboratory to generate EnS cell line with inducible, stable knock-down (kd) for Sdc-1 named KdS1 as published before [25]. Furthermore, HTR8/SVneo (ATCC® CRL3271™) immortalized first-trimester trophoblast cells were used [30].

### 2.2. Cell Culture

Both endometrial cell lines were maintained at 37°C and 5% CO_2_ in a mixture of 3/4 (v/v) DMEM high glucose and 1/4 (v/v) MCDB 105 (both Biowest, Nuaillé, France). The cell-culture media were supplemented with 10% (v/v) charcoal-stripped fetal bovine serum (FBS), 1 × penicillin/streptomycin, 40 *μ*g/mL gentamycin, 5 *μ*g/mL insulin (Sigma-Aldrich, Steelze, Germany), 2 mM L-glutamine, 1 mM nonessential amino acids, and 1 × sodium pyruvate (all except insulin Biowest). KdS1 cells were cultured in presence of two selection antibiotics: 6 *μ*g/mL blasticidin and 200 *μ*g/mL Zeocin (both InvivoGen, San Diego, CA, USA). HTR8/SVneo were cultured in RPMI 1640 supplemented with 5% (v/v) FBS and 1 × penicillin/streptomycin (Biowest).

### 2.3. Decidualization

The decidualization was introduced by applying 0.5 mM 8-Br-cAMP (8-bromoadenosine-cyclic monophosphate) (Biolog, Bremen, Germany), 1 *μ*M MPA (medroxyprogesterone acetate) (Sigma-Aldrich), 50 *μ*g/mL ascorbic acid, and 10 *μ*g/mL transferrin in insulin-free minimal medium (DMEM with 2% FBS) for 72 h [31, 32]. Decidualization was proven by amplification of the decidualization marker prolactin (PRL) using standard polymerase chain reaction (PCR) with an amplification product size of 250 bp (as shown in Table 1).

### 2.4. Spheroid Formation via Hanging Drop and Confrontation

3 × 10^4^ St-T1 or Tet-induced KdS1 cells and 2 × 10^4^ HTR8/SVneo cells per 30 *μ*L culture media were separately plated on the lid of a Petri dish (see Figure 7). The bottom of the dish was prefilled with PBS and the lid inverted after plating. The droplet cultures were incubated for 48 h for spheroid formation before staining with 1 *μ*m MitoTracker® (Invitrogen Thermo Fisher Scientific, Waltham, MA, USA) either green (M7514) or orange (M7510) for 30 min at 37°C and 5% CO_2_ (see Figure 7).

After staining, the spheroids (either St-T1 with HTR8/SVneo or KdS1 with HTR8/SVneo; 2 St-T1 spheroids served as control) were cultured in confrontation for 2–48 h and photographed with a confocal microscope (Zeiss LSM 510 META, Carl Zeiss, Jena, Germany). Measurement of center-to-center distances was performed applying the ImageJ software version 1.49 [33].

### 2.5. Experimental Conditions

St-T1 and KdS1 were decidualized and incubated with 1 *μ*g/mL tetracycline (Tet) for Sdc-1 kd induction (only KdS1 and dKdS1) after reaching 70% confluence and incubated with 10, 25, and 50 *μ*M MAPKK (MEK1/2) inhibitor (PD 98.059, Sigma-Aldrich) and 0.1, 0.5, 1, 5, 10, and 25 *μ*M JNK inhibitor (AEG 3482, Tocris, Bristol, UK) for 2 h at 37°C and 5% CO_2_ followed by 48 h of incubation with 10 ng/mL IL-1*β* for St-T1 (data not shown) and KdS1 and 0.1 ng/mL IL-1*β* for dSt-T1 and dKdS1 at equal conditions [20]. Afterwards, the cell-culture media supernatants were harvested and analyzed regarding the CXCL1 expression by ELISA. RNA and proteins were isolated from treated cells. St-T1, KdS1, dSt-T1, and dKdS1 cells with IL-1*β* coincubation but without inhibitor pretreatment served as controls. The inhibitors were previously diluted with DMSO (Sigma-Aldrich). The DMSO tolerability of the cells was tested before experimental performance (data not shown).

For Western blot analysis of pERK and p-c-Jun activity the non-d and d cells were treated with IL-1*β* (concentrations seen above) for 15 min followed by protein isolation. Corresponding cells without IL-1*β* incubation served as control. dSt-T1 and dKdS1 were incubated with 0.1 ng/mL IL-1*β* for 48 h for determination of FZD4 and FZD6, beta-catenin phosphorylation, MMP7 expression, and (NF*κ*B) activity. Treatment with 5 ng/mL TNF*α* served as control for NF*κ*B activity.

For the QuantiGene Plex Assay KdS1 and dKdS1 were incubated with 1 *μ*g/mL Tet and dSt-T1 cells were incubated with 0.1 ng/mL IL-1*β* for 48 h at 37°C and 5% CO_2_ followed by sample preparation according to the manufacturer' protocol (Affymetrix Panomics, Santa Clara, CA, USA).

All experiments were conducted with 3 technical repeats and 3-4 biological repeats per condition.

### 2.6. RNA Isolation, Reverse Transcription, and PCR

Total RNA was isolated using peqGOLD TriFast™ (PEQLAB, Erlangen, Germany). This method is based on the single step RNA isolation described by Chomczynski and Sacchi [34]. After quantification, 2 *μ*g total RNA were used for preparing DNA-free RNA through desoxyribonuclease I digestion (DNase I, Thermo Fisher Scientific) and a reverse transcription PCR was performed by applying the High Capacity© cDNA archive kit (Applied Biosystems Inc., Foster City, CA, USA), and both steps were carried out according to the manufacturers' protocol.

The housekeeping gene *β*-actin was amplified for all performed PCRs as control (Figure 2). The reactions consisted of 1 × DreamTaq™ Green PCR Master Mix (Thermo Fisher Scientific), 1 and, respectively, 2 *µ*l cDNA (for ß-actin and, resp., PRL PCR) cDNA from the reverse transcription, 0.3 *μ*M forward and reverse primer, and dH_2_O ad 25 *μ*L. The PCRs were carried out in a peqSTAR 96 universal gradient thermocycler (PEQLAB). The according primer sequences are shown in Table 1. PCR products were immediately separated electrophoretically in a 1% agarose gel in presence of the nucleic acid gel stain GelRed™ (1 : 10.000, Biotium, Hayward, USA) or stored at −20°C for subsequent electrophoresis. After separation the agarose gel was analyzed by the GelDoc 1000 system (Bio-Rad Laboratories, Hercules, USA).

### 2.7. Protein Isolation, Western Blot, and Dot Blot Analysis

Total protein was isolated using peqGOLD TriFast (PEQLAB). Proteins were resuspended with 1% SDS-solution and supplemented with phosphatase inhibitor cocktails B and C (Santa Cruz Biotechnology Inc., Santa Cruz, CA, USA) and protease inhibitor complete (Roche Diagnostics, Penzberg, Germany) according to the manufacturer's instructions. Protein concentration was measured with a Pierce BCA Protein Assay Kit (Thermo Fisher Scientific).

For SDS-PAGE, 30 *μ*g protein of each sample was filled up with 5x sample loading buffer (375 mM Tris-HCl pH 6.8, 60% glycerol, 0.3% (w/v) SDS, and 1,5% bromophenol blue) and separated electrophoretically through a 10% SDS-gel with a ratio of 29 : 1 acrylamide : BIS-acrylamide. After SDS-PAGE the proteins were transferred to an Immobilon™ polyvinylidene fluoride (PVDF) membrane with a pore diameter of 0.45 *μ*m (Merck Millipore, Darmstadt, Germany). For dot blot analysis, 30 *μ*g total protein was spotted on the PVDF membrane followed by complete drying. Afterwards, the membranes were incubated with 5% fat-free dry milk (Carl Roth, Karlsruhe, Germany) solution in Tris buffered saline with 0.1% Tween 20 (AppliChem, Darmstadt, Germany) for 1 h at room temperature (rt) for blockage of nonspecific binding. The following primary antibodies were used in this study: rabbit monoclonal anti-human p44/42 MAPK (ERK1/2, 42 and 44 kDa, 1 : 2000 dilution), rabbit monoclonal anti-human phospho-p44/42 MAPK (phospho-ERK1/2 (Thr202/Tyr204) (pERK), 42 and 44 kDa, 1 : 2000 dilution), rabbit monoclonal anti-human c-Jun (43 and 48 kDa, 1 : 500 dilution), rabbit monoclonal anti-human phospho-c-Jun ((Ser73) (pc-Jun), 48 kDa, 1 : 500 dilution, all Cell Signaling Technology, MA, USA), rabbit polyclonal anti-human Sdc-1 (34 kDa, 1 : 1000 dilution, Abcam), rabbit polyclonal anti-frizzled 4 (60 kDa, 1 : 2000 dilution, Abcam), rabbit monoclonal anti-frizzled 6 (79 kDa, 1 : 500 dilution, Cell Signaling), rabbit polyclonal anti-beta-catenin (96 kDa, 1 : 1000 dilution, Abcam), rabbit polyclonal anti-phospho-beta-catenin (86 kDa, 1 : 800 dilution, Abcam), rabbit polyclonal anti-MMP7 (28 kDa, 1 : 500 dilution, Abcam), rabbit monoclonal anti-NF*κ*B p65 (65 kDa, 1 : 1000 dilution), rabbit monoclonal anti-phospho NF*κ*B pp65 (65 kDa, 1 : 1000 dilution), and mouse monoclonal anti-I*κ*B*α* (39 kDa, 1 : 1000 dilution, all Cell Signaling). The primary antibodies were incubated overnight at 4°C. The membranes were washed 3 times with TBS/0.1% Tween 20 before the secondary antibodies, and either anti-rabbit IgG (1 : 2000 dilution) or anti-mouse IgG antibodies (1 : 2000 dilution, R&D Systems, Minneapolis, MN, USA) all conjugated to horseradish peroxidase (HRP) were applied for 1 h at rt. After 3 washes with TBS/0.1% Tween 20, proteins were visualized by chemiluminescence activated by Clarity™ Western ECL Substrate (Bio-Rad). Protein band sizes were determined using Prestained Protein Molecular Weight Marker (Thermo Fisher Scientific). *β*-Actin western blots served as controls (mouse monoclonal anti-human, 42 kDa, 1 : 2000 dilution, Abcam). Evaluation of the pixel density was performed with the image processing program ImageJ 1.46r [33].

### 2.8. CXCL1 ELISA

CXCL1 was measured by ELISA (R&D Systems Duo Set) in cell-culture supernatant according to the manufacturers' protocol. Briefly, supernatant was diluted 40 times and measured in duplicate. After coating the 96-well plate with 4 *μ*g/mL CXCL1 capture antibody overnight at rt and blocking of unspecific antibody binding sites for 1.5 h at rt, the diluted supernatants and standard solutions were applied followed by 2 h incubation. Subsequently incubation with 50 *μ*g/mL detection antibody for 2 h, streptavidin-HRP solution for 20 min, substrate solution for 20 min, and stop solution was performed. Evaluation was performed using a microplate reader with 450 nm wavelength (PerkinElmer Victor2, Waltham, MA, USA).

### 2.9. QuantiGene Plex Assay (QGP Assay)

QGP assay was used to determine the Sdc-1 mRNA expression in all endometrial cell lines used in this study according to manufacturers' protocol. Briefly, after sample preparation through cell lysis the samples were mixed with the bead mix and were applied on the 96-well plate followed by light protected incubation for 18–22 h at 56°C on an orbital shaker. Subsequently, 4 signal amplification steps and a signal detection step were accomplished. Experiments were carried out in triplicate. Analysis was carried out with a Luminex© detection system (Bio-Rad Laboratories).

### 2.10. Statistical Analysis

The statistical significance of the RNA and protein expression and center-to-center distance measurement were calculated by applying the Student's *t*-test with *p* < 0.05.

## 3. Results

### 3.1. Validation of Sdc-1 kd

**Figure 1:**
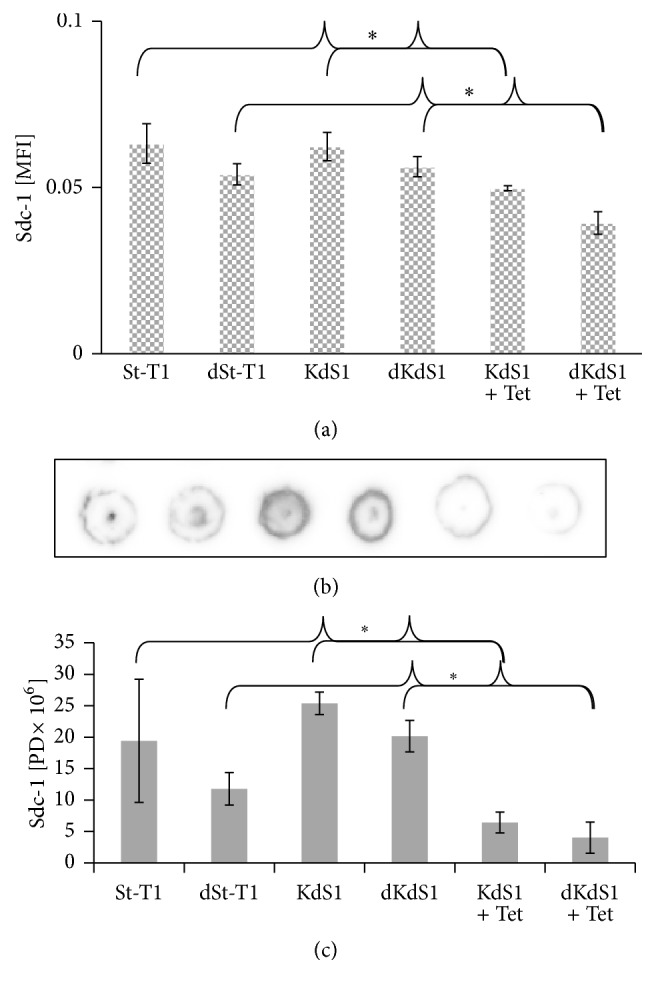
Evaluation of the Sdc-1 kd in the human EnS cell lines. (a) Sdc-1 mRNA levels determined by QGP assay [MFI = mean fluorescence intensity], the qualitative Sdc-1 protein expression was determined by dot blot analysis depicted in (b) and the corresponding pixel density evaluation is shown in (c) with and without decidualization (+/−d) and with and without inducing the Sdc-1 kd (+/−Tet) in KdS1 cells with 1 *μ*g/mL Tet. The dot blot analysis was performed with 30 *μ*g protein per dot, and samples were applied in the same order as in (c). Data represent means ± SD with ^*∗*^*p* < 0.05 for Tet treated KdS1 cells versus untreated KdS1 (+/−d) cells.

Before starting sample preparation, Sdc-1 kd in the EnS cell line KdS1 via Tet incubation as well as Sdc-1 expression in St-T1 was proven on mRNA and protein level by QGP assay (Figure 1(a)) and dot blot (Figures 1(b) and 1(c)). Sdc-1 kd was induced through applying 1 *μ*g/mL Tet in KdS1 and St-T1 and KdS1 cells were then decidualized for 72 h. Cell lysates were taken for QGP assay and total protein was isolated for dot blot analysis. Both Sdc-1 mRNA (Figure 1(a)) and protein were significantly decreased after inducing the Sdc-1 kd in KdS1 and dKdS1 (Figures 1(b) and 1(c)).

### 3.2. Validation of Decidualization

**Figure 2:**
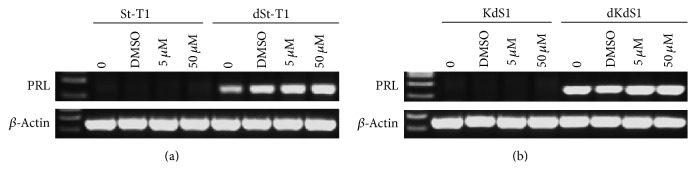
Representative qualitative PRL expression in St-T1 (a) and KdS1 (b) samples before and after decidualization (dSt-T1, dKdS1). Samples were treated only with IL-1*β* (0), with IL-1*β* and DMSO (DMSO) and with IL-1*β* and MEK1/2 inhibitor (5 *μ*M, 50 *μ*M) as indicated. Expression of *β*-actin served as housekeeping control.

PRL mRNA expression by PCR was analyzed for all samples examined. PRL amplification was only detected in decidualized cells. The representative result shows the PRL amplification in IL-1*β* and MEK1/2 inhibitor treated St-T1, dSt-T1, KdS1, and dKdS1 cells (Figures 2(a) and 2(b)).

### 3.3. Western Blot Analysis of MAPK Signaling

**Figure 3:**
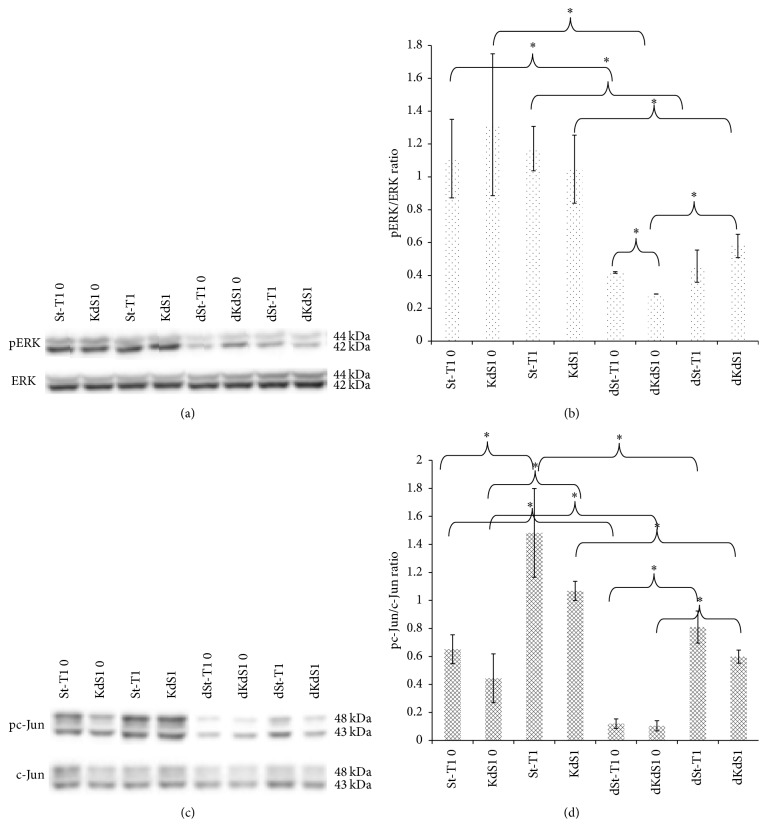
Protein expression analysis of ERK/pERK (44, 42 kDa) and c-Jun/pc-Jun (48, 43 kDa), in St-T1 and the Sdc-1 kd cell line KdS1 (+/−d). (a) shows the protein expression of ERK and pERK in St-T1, dSt-T1, KdS1, and dKdS1 after treatment with IL-1*β* for 15 min compared to controls without IL-1 *β* treatment in a representative western blot, in (b) the corresponding pixel density evaluation as pERK/ERK ratio, in (c) the c-Jun and pc-Jun expression in St-T1, dSt-T1, KdS1, and dKdS1 after treatment with IL-1*β* for 15 min compared to controls without IL-1 *β* treatment in a representative western blot, and in (d) the corresponding pixel density evaluation as pc-Jun/c-Jun ratio. Data represents means ± SD with ^*∗*^*p* < 0.05. GAPDH served as control (data not shown).

All cells showed a steady synthesis of ERK. There was no significant increase of phosphorylation upon incubation with IL-1*β*. On the contrary, the pERK levels and the pERK/ERK ratios were statistically significantly higher in non-dSt-T1 and Tet-induced KdS1 than in the decidualized counterparts after 15 min of IL-1*β* incubation (Figures 3(a) and 3(b)). The pERK/ERK ratio was lower in dKdS1 control compared to dSt-T1 control. Incubation with IL-1*β* resulted in a significant increase in ERK phosphorylation. Therefore, the level of phosphorylation of ERK depended primarily on decidualization but not on the Sdc-1 expression (Figure 3(b)).

IL-1*β* incubation led to a statistic significant increase of pc-Jun/c-Jun ratio in non-dSt-T1 and KdS1. A significant decrease of the pc-Jun level and pc-Jun/c-Jun ratio was detected upon decidualization of St-T1 and KdS1 (Figures 3(c) and 3(d)). Furthermore, the reduction of Sdc-1 on the surface of dKdS1 led to a significant lower activation of c-Jun than in dSt-T1 (Figure 3(d)). As stated above, the effect of lower phosphorylation levels seems to depend on decidualization status of the cells. Il-1*β* signal tends to be mediated preferably via c-Jun signaling in EnS.

### 3.4. ELISA Analysis of CXCL1 Protein Secretion

**Figure 4:**
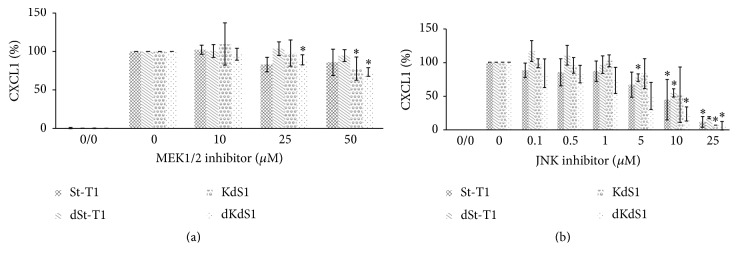
Quantitative analysis of CXCL1 protein secretion (%) of human EnS cell lines St-T1 and KdS1 measured by ELISA. (a + b) St-T1, dSt-T1, KdS1, and dKdS1 were treated with indicated concentrations of the inhibitors for 2 h, and subsequently the cells were incubated with IL-1*β* for 48 h (10 ng/mL for nondecidualized and 0.1 ng/mL for decidualized cells). Shown are means ± SD with ^*∗*^*p* < 0.05 for treated cells versus untreated cells. Cells with no inhibitor but IL-1*β* treatment served as controls (0) (=100% CXCL1 secretion); 0/0 cells (no inhibitor, no IL-1*β*) served as secretion controls (=0%).

A significant decrease of CXCL1 secretion was detected by a starting dose of 25 *μ*M MEK1/2 inhibitor in dKdS1 and 50 *μ*M in KdS1 and dKdS1 cells (Figure 4(a)). Neither St-T1 nor dSt-T1 responded to MEK1/2 inhibitor incubation regarding CXCL1 expression (Figure 4(a)). Therefore Sdc-1 seems to be involved in the MEK pathway.

The incubation with JNK inhibitor led to a statistically significant reduction in CXCL1 protein expression in dSt-T1 starting at 5 *μ*M, in St-T1 and dKdS1 starting at 10 *μ*M, and in all cell types examined at 25 *μ*M JNK inhibitor (Figure 4(b)). The JNK signaling cascade seems to be pivotal in EnS with and without decidualization. In addition, Sdc-1 depletion revealed no statistically significant effect regarding JNK signaling.

### 3.5. Western Blot Analysis of Wnt Components, MMP7 and NF*κ*B Signaling

**Figure 5:**
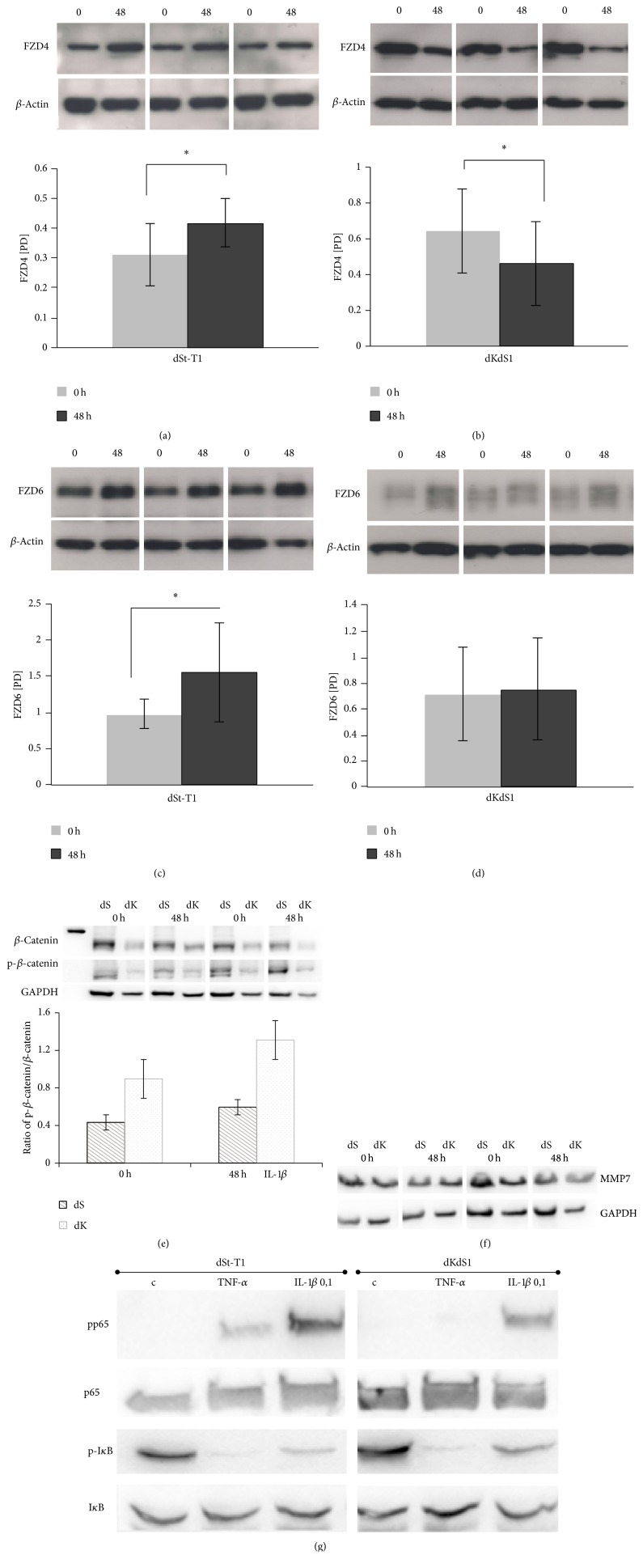
Western blot protein expression analysis of FZD4 (60 kDa), FZD6 (75 kDa), phospho- (86 kDa) and holo-*β*-catenin (96 kDa), and MMP7 (28 kDa) in decidualized St-T1 and KdS1. (a) shows the protein expressions of FZD4 in dSt-T1 and (b) in dKdS1, (c) of FZD6 in dSt-T1 and (d) in dKdS1, (e) of *β*-catenin and phospho-*β*-catenin, and (f) of MMP7 and (g) NF*κ*B signaling after incubation with 0.1 ng/mL IL-1*β* for 0 and 48 h in representative western blot analysis and the corresponding pixel density evaluation (omitted for MMP7, p65, and I*κ*B; TNF*α* was used as control for NF*κ*B activity). Data represents means ± SD with ^*∗*^*p* < 0.05 for IL-1*β* treated cells versus untreated cells. *β*-Actin analysis served as control.

FZD4 protein expression was significantly decreased in dKdS1 compared to the significant increase in dSt-T1 cells after IL-1*β* incubation for 48 h (Figures 5(a) and 5(b)).

The protein expression of FZD6 showed also a significant increase in dSt-T1 after incubation with IL-1*β* and no effect on the FZD6 expression in dKdS1 (Figures 5(c) and 5(d)). Regarding phosphorylation of beta-catenin, low levels could be detected for the ratio in dST-T1 with and without incubation with IL-1*β* for 48 h whereas dKdS1 expressed generally higher levels of phosphorylated beta-catenin without reaching statistical significance upon IL-1*β* incubation (Figure 5(e)). As Wnt signaling upon beta-catenin is linked with MMP7 activity, protein expression was measured in dSt-T1 and dKdS1 before and after IL-1*β* incubation (Figure 5(f)). The level of MMP7 expression did not change upon IL-1*β* treatment. Furthermore, there was no difference between dSt-T1 and dKdS1 regarding MMP7 expression (Figure 5(f)). Due to the complexity of signaling networks, NF*κ*B (p65) was further qualitatively examined (Figure 5(g)). There were no differences regarding p65 and I*κ*B*α* expression between dSt-T1 and dKdS1 with and without incubation with TNF*α* or IL-1*β*. Interestingly, pp65 could not be observed in dKdS1 incubated with TNF*α* but could be detected with IL-1*β* (Figure 5(g)). dSt-T1 showed a higher expression of pp65 after IL-1*β* incubation than with TNF*α* (Figure 5(g)). Higher levels of pI*κ*B could be detected in control cells than in TNF*α* or IL-1*β* incubated cells with and without knock-down of Sdc-1 (Figure 5(g)).

### 3.6. Spheroid Formation and Confrontation of Endometrial Cells and Trophoblast Cells

**Figure 6:**
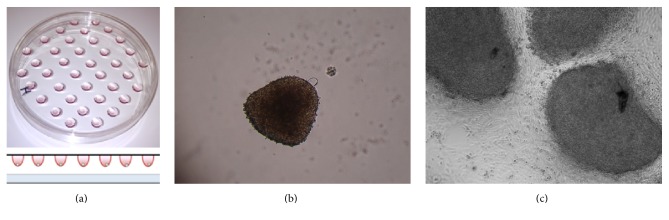
Photograph and schematic diagram of the hanging drop culture model (a). 100x magnification of a HTR8/SVneo spheroid (b) and attachment of HTR8/SVneo spheroids on a 2D St-T1 cell layer (100x magnification) (c).

**Figure 7:**
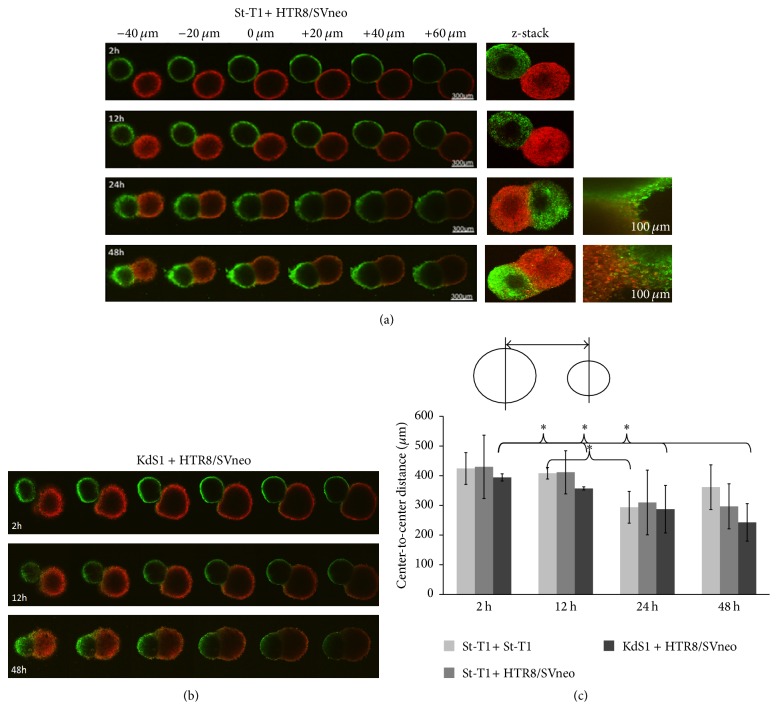
Representative confocal microscopy of St-T1 (a) and KdS1 (b) spheroids (green) cocultured with HTR8/SVneo spheroids (red) incubated for 0–48 h imitating human preimplantation and implantation processes. Measurement of center-to-center distances [*μ*m] (c) was chosen to examine 3D attachment and invasion. Data represents means ± SD with ^*∗*^*p* < 0,05 for trophoblast-endometrium incubated cells versus control (2 St-T1 spheroids).

The endometrial stromal cell spheroids from St-T1 as well as Tet-induced KdS1 and the trophoblast HTR8/SVneo spheroids could be visualized macroscopically after 48 h of culture in a hanging droplet (Figures 6(a) and 6(b)). Furthermore, all the spheroids could be handled easily by pipetting. In a previous attempt, HTR8/SVneo spheroids were applied on a St-T1 monolayer without Matrigel sublayer (Figure 6(c)). Attachment could be visualized, but deep invasion or cell interaction could not be measured appropriately due to the dense composition of the spheroids. On that account, the rate of interaction between maternal and trophoblast cells was measured via calculation of center-to-center distances after 0–48 h incubation time in the current experiments (Figures 7(a)–7(c)). Comparing the cellular interaction and invasion between either St-T1 or KdS1 and HTR8/SVneo, the most intense and statistically significant interaction could be observed for KdS1 with HTR8/SVneo after a minimum of 12 h (Figure 7(c)).

## 4. Discussion

A well balanced implantation process composed of a sensitive and complex network of numerous different ligands, receptors, transcription factors, and signaling pathways on the maternal as well as on the embryonic side is the fundament of a healthy pregnancy. In spite of gaining more data of the molecular processes at the human embryo-maternal interface, the in vivo process of implantation still remains a black box in human reproduction. Up-to-date gene and protein profiles of decidualized human endometrium of healthy volunteers and patients undergoing assisted reproduction are available, but all of them are limited regarding approach and technique [35–38]. Therefore, artificial cell-culture models are still required to imitate the uterine environment as physiologically as possible. Furthermore, due to legal restrictions in many countries a good substitute for the embryo is substantial.

Chemokine ligands, like CXCL1, play a crucial role at the embryo-maternal interface and an intensive upregulation of CXCL1 expression was shown in dEnS cells induced by various embryo derived and immune cell media before [17, 18, 39–41]. In the female reproductive tract CXCL1 is expressed throughout the cycling endometrium, mainly in the stroma with increasing amounts during the secretory phase, and particularly decidualization enhances the expression of CXCL1 in EnS cells [42]. Sdc-1, the coreceptor of CXCL1, is a heparan sulfate proteoglycan cell surface molecule which is localized on endometrial stromal cells, myometrium under pregnant conditions, placenta, and amniotic epithelium [20, 43, 44]. Known signaling pathways involved in the human implantation process are the MAPK and Wnt pathway, both playing roles in differentiation, proliferation, angiogenesis, and apoptosis at the embryo-maternal interface [2, 13].

The present study investigated the influence of Sdc-1 on CXCL1 expression through the ERK1/2 and JNK signaling pathway and on the expression of the Wnt signaling receptors, FZD4 and FZD6, and Wnt mediator beta-catenin in human endometrial stromal cells. The immortalized EnS cell line St-T1 and the EnS cell line KdS1, derived from St-T1, displaying a Sdc-1 kd were used for all experiments. Successful decidualization was proven by a qualitative PRL PCR. Furthermore, the EnS cells were treated with the embryonic secretion product IL-1*β* as a substitute for the ethically inaccessible healthy embryo or human embryonic stem cells. IL-1*β* was shown to be an induction factor for CXCL1 expression before [20, 42]. Both investigated MAPK signaling pathways, ERK1/2 and JNK, are known to regulate CXCL1 expression in different cell types [42, 45]. For this purpose, nondecidualized naïve EnS and dSt-T1 and Tet-induced KdS1 cells were treated with IL-1*β* for ERK1/2 and c-Jun phosphorylation studies. Furthermore, cells were incubated with specific inhibitors for MEK1/2 and JNK and subsequently treated with IL-1*β* for CXCL1 expression studies. The loss of Sdc-1 seems to have no effect on the signal transduction, since the investigated phosphorylation of ERK1/2 showed no alterations between presence and depletion of Sdc-1 in EnS and dEnS cells. However, it seems that it is rather the differentiation state of the EnS, especially the decidualization that affects the amount of phosphorylation of ERK and c-Jun. This data is supported by similar findings of Yoshino and coworkers reporting a less intense phosphorylation of MAPK p38 after decidualization of EnS cells [46].

In the light of the current literature, the influence of Sdc-1 on the regulation of signaling pathways seems to be cell-type specific. Our analysis of the c-Jun phosphorylation uncovered a reduced c-Jun activation in dKdS1 cells after inducing the Sdc-1 kd compared to dEnS and therefore a direct influence of the surface molecule Sdc-1 on JNK signaling modulated by decidualization. In vivo, the decidualization process is followed by a secretory transformation of the uterine glands, differentiation of the stromal cells, influx of uterine natural killer cells, and vascular remodeling of the endometrial stromal compartment [47]. This structural reshaping is accompanied by an altered gene transcription, which has probably an effect on c-Jun phosphorylation by modulating the signal transduction dependent on Sdc-1. Here, the direct upstream regulators of c-Jun, MKK4/7 and JNK, could potentially be the targets of Sdc-1. The experiment shown within this manuscript uncovers that CXCL1 regulation is Sdc-1 dependent via MEK, but not via JNK signaling. This outcome suggests a sensitization of the EnS and dEnS cells towards the MEK1/2 inhibitor by depletion of Sdc-1.

Data regarding Sdc-1's influence on signaling pathways are limited; Sun et al. examined the inhibition of MEK mediated by an upregulation of Sdc-1 through docosahexaenoic acid in human breast cancer cells and in mouse mammary cells initiating apoptosis in these cells, and Lei et al. detected that Sdc-1 overexpression inhibits p38 MAPK pathway in a rat model of myocardial infarction [48, 49]. Rapraeger et al. focused on Sdc-1's role as an essential coreceptor in a core complex during angiogenesis in endothelial cells [50]. In addition, progression of multiple myeloma is positively correlated with Sdc-1 expression on malignant plasma cells. Sdc-1 interacts with insulin-like growth factor 1 receptor and integrins enabling angiogenesis and cell survival hence blocking of Sdc-1 via synstatin might result in new therapeutic approaches in cancer treatment [51–53]. Concerning embryo development, Sdc-4 regulates the WNT/JNK pathway during foregut development in* Xenopus* [54]. Since those two studies deal with the effect of Sdc-1 overexpression we can only hypothesize that the kd of Sdc-1 might therefore activate MAPK. But it seems to be the contrary regarding CXCL1 expression in our model of EnS which might reflect a cell-type specific function of Sdc-1.

The Wnt signaling pathway plays a pivotal role during embryo development, implantation preparation, and early pregnancy on the maternal side [2, 13]. Since the expression of FZD4 and FZD6 and their potential role in regulating the implantation process were already demonstrated in the mouse and ovine model [55, 56], the present study was supposed to clarify the expression in human decidua imitating implantation on the one hand and the possible relation to Sdc-1 on the other hand. Herein it was shown for the first time in a human decidualized stromal cell line that incubation with the embryo surrogate IL-1*β* led to a significant increase of FZD4 as well as FZD6 expression on protein level in dEnS cells. Interestingly, after kd of Sdc-1, incubation with IL-1*β* had an opposite effect on the FZD4 expression and no effect on FZD6. The increase of FZD4 and FZD6 expression in dEnS indicates a possible important role of Wnt signaling at the embryo-maternal interface in human. The Wnt signaling activation seems to be conserved in mammalian embryo development connecting maternal endometrium and embryo trophoblast [52]. Moreover, FZD4 might have an important function for the corpus luteum and therefore progesterone production, because FZD4 ko mice are infertile [57]. Regarding angiogenesis as another important issue at the embryo-maternal interface, it was found that FZD4 is regulated by Norrin and essential for retinal angiogenesis and embryo development [58]. As there are no data available connecting Sdc-1 and FZD4 so far we hypothesize that the decrease of FZD4 in dKdS1 cells could be based on the autocrine regulation of Wnt signaling and NF*κ*B signaling. Our preliminary data suggest that the MMP7 expression seems to be independent of Sdc-1 in dEnS. In case of a direct interaction between MMP7 expression and Sdc-1 presence we would have assumed a joint expression scheme, namely, a higher expression during secretory phase and a lower expression during proliferative phase. In vivo data of MMP7 expression during the proliferative phase of the cycle and during menses also underline separate actions of MMP7 and Sdc-1 in EnS as also observed in our study [59]. Furthermore, new data on transcriptomic profiling suggest a different expression of MMP7 between fertile and infertile females [60].

The phosphorylation of beta-catenin results in the degradation of the protein. Our data showed a higher ratio of p-beta-catenin after 48 h of incubation in dKdS1 with IL-1*β* versus dSt-T1. In the literature beta-catenin expression referred to an estrogen-dependent action of Wnt signaling. The localization of beta-catenin in endometriotic lesions underlines a role during neovascularization via VEGF [61, 62]. As angiogenesis is important for embryo implantation and nutrition, a regular Sdc-1 expression might therefore ensure this capacity. Up to date, intracellular Sdc-1 action could be focused on the influence on the transcription factor NF*κ*B (for review [63]). In our qualitative analysis, we did not see a different expression of NF*κ*B family members between dSt-T1 and dKdS1 after 48 h of incubation. It might be possible though that another member of the NF*κ*B family is influenced by Sdc-1 during embryo implantation in EnS, for example, I*κα*, which was not part of this study (for review [64]).

In addition to the expression analysis of Wnt signaling factors, we aimed to imitate a cellular interaction between endometrial and trophoblast cells using a new 3D spheroid model. HTR8/SVneo trophoblast cells have been extensively examined and spheroid formation was published before [65, 66]. In early experiments, we first tried to coincubate preformed HTR8/SVneo spheroids with adherent EnS. Due to the impossibility of measuring the invasion of both cell types properly on a plain dish, we started to generate spheroids from EnS cells trying to mimic a physiological 3D embryo-maternal environment. Applying this model, we were able to examine the intercellular distances between EnS with and without Sdc-1 kd and HTR8/SVneo trophoblast cells for up to 48 h under a confocal microscope. The most intense interaction could be shown for KdS1 and HTR8/SVneo cells. Weber et al. conducted a comparable experiment with HTR8/SVneo spheroids and placenta villi of caesarean sections showing a repopulation of the chorionic villi [66]. Based on the fact that Sdc-1 kd cells and HTR8/SVneo showed the strongest interaction, it seems that Sdc-1 acts as a regulator of the invasion depth at the trophoblast-decidual interface most likely by influencing apoptotic abilities and cellular motility [26, 67]. Bearing in mind that Sdc-1 can act via its ectodomain as a coreceptor and its endodomain can interact with integrins, the current findings underline its high potential for the regulation of chemokine-based communication, cell survival, motility, and invasion in different physiological and pathological settings [25, 26, 53, 67]. In a prospective clinical approach a higher expression of Sdc-1 on chorionic villi could be positively related to a mature baby at birth and a reduced risk of premature birth. Furthermore, low expression of Sdc-1 was found on chorionic villi of smoking mothers [68].

Based on these findings we hypothesize that Sdc-1 is a marker for implantation competence of the decidualized endometrium in women with unexplained implantation failure or pregnancy-disorders based on an insufficient implantation and angiogenesis.

## 5. Conclusion

The implantation of a human embryo into the maternal endometrium is a complex and tight controlled network of chemokines, their corresponding receptors, and signaling pathways. Sdc-1 plays an important role in MAPK mediated signaling of CXCL1 expression and cell invasion. Both processes are essential for a proper embryonic implantation. Therefore, Sdc-1 could act as a diagnostic marker for implantation failure or disorder.

## Figures and Tables

**Table 1:** PCR primers for *β*-actin and PRL and their amplicon size.

Primer	Sequence 5′ → 3′	Amplicon size [bp]
*β*-Actin	5′-CGGGACCTGACTGACTACC-3′	239
	3′-AGGAAGGCTGGAAGAGTGC-5′	

PRL	5′-GCTTCTGTATCATCTGGTCACG-3′	247
	3′-TGCGTAGGCAGTGGAGCAG-5′	

## Abbreviations

JNK: C-Jun N-terminal kinase
CXCL: CXC motif ligand
CXCR: CXC motif receptor
d: Decidualized
DMEM: Dulbecco's modified Eagle's medium
EnS: Endometrial stromal cells
ERK: Extracellular kinases
FBS: Fetal bovine serum
FZD: Frizzled
HTR8/SVneo: Human trophoblast cell line immortalized with SV40 virus
IL-1*β*: Interleukin-1 beta
KdS1: EnS cell line with an inducible, stable knock-down for Sdc-1
kd: Knock-down
ko: Knock-out
MAPK: Mitogen-activated protein kinases
MAPKK, MKK: MAPK kinase
MPA: Medroxyprogesterone acetate
MCDB: Molecular, cellular, and developmental biology
NF*κ*B: Nuclear factor kappa B
PCR: Polymerase chain reaction
PRL: Prolactin
QGP assay: QuantiGene Plex Assay
RPMI: Roswell Park Memorial Institute
rt: Room temperature
St-T1: Human endometrial stroma cell line immortalized with SV40 virus
Sdc-1: Syndecan-1
Tet: Tetracycline
TNF*α*: Tumour necrosis factor alpha
Wnt: Wingless-Int1s.
